# Midwifery

**Published:** 1838-04

**Authors:** 


### MIDWIFERY.

### *Spontaneous Amputation of the Limbs in Utero.* By Richard Smith, Senior-Surgeon, Bristol Infirmary.

That limbs are so separated from the body of the fœtus can scarcely be controverted after the printed statements of Drs. Montgomery and Simpson. The *quo modo*, however, the manner in which the occurrence takes place, is not quite so clear; and I perceive that the question has been mooted, particularly by Dr. Robert Lee. Having, in my museum at the Infirmary of Bristol, a preparation which, I think, may throw some light upon the matter, I feel it a duty to address these lines to you. It was sent to me by my former pupil and friend, Mr. Charles Bleeck, Surgeon, of Warminster, Wilts. In 1835 that gentleman attended a female with her second child. It was a breech presentation, and, in due course, the lower parts and trunk up to the axilla, were expelled. Mr. B. proceeded to bring down one of the arms, experiencing no difficulty, but the other resisted all his efforts; it was, in fact, so immoveably fixed, that after using as much force as he felt justified in doing, he desisted; in about ten minutes after this a violent pain brought into the world the head, the arm, and the whole of the secundines at the same moment; the child was alive; the cause of the resistance was now manifest. From the upper part of the funis, the circulation of which was not at all impeded, passed a very strong and tough band, about an inch and a half long, with its end firmly attached to the surface of the placenta; this band was perforated, and grasped, in the aperture, the arm so tightly just above the elbow, that the soft parts were, if I may be allowed the expression, cut down to the bone; the limb was enlarged to four times its usual bulk; the arm was liberated from the band, and the child appeared at first to be going on well, but in twenty-four hours it became restless and uneasy; the limb, too, grew hot, put on a livid appearance, and was soon covered with vesications; the vitality of the limb being apparently at an end at the expiration of forty-eight hours, Mr. Bleeck deemed it advisable to disengage it; this was easily effected, and not the slightest hemorrhage followed, although the axillary artery continued to beat slowly. The child seemed conscious of relief from its incumbrance, and quiet during thirty-six hours, at the end of which time it was seized with convulsions, and died. Now, Sir, my view of the matter is this,—I consider that at an early period of utero-gestation a band of coagulable lymph had been thrown out from the funis to the neighbouring part of the placenta; in closing or being coiled around the arm of the fœtus, that this band became vascular, thickened, and tough; thus circumstanced, it would yield but imperfectly to the growth of the limb, but rather continued to grasp it more and more firmly, and thus, by its continued pressure, produced absorption of the soft parts actually down to the bare humerus. It is easy to imagine how easily a struggle of the child might snap off the latter, and thus circumstanced it would probably have been the case in the present instance, but that the period of utero-gestation was at end, and that the bone formed as yet an attachment to the body. If I am right in my conjectures the above forms an easy solution to the marvellous riddle, and if it may happen thus in one instance, it may occur in several.

*Lancet. February* 17, 1838.

